# A guide to epigenetics in leukaemia stem cells

**DOI:** 10.1002/1878-0261.13544

**Published:** 2023-11-02

**Authors:** Shuchi Agrawal‐Singh, Jaana Bagri, Nathalie Sakakini, Brian J. P. Huntly

**Affiliations:** ^1^ Department of Haematology, Jeffrey Cheah Biomedical Centre University of Cambridge UK; ^2^ Cambridge Stem Cell Institute University of Cambridge UK; ^3^ Haematology Service Cambridge University Hospitals UK

**Keywords:** epigenetic plasticity, heterogeneity, leukaemia stem cells, myeloid neoplasms, single cell studies and multiomics, targeting LSC

## Abstract

Leukaemia stem cells (LSCs) are the critical seed for the growth of haematological malignancies, driving the clonal expansion that enables disease initiation, relapse and often resistance. Specifically, they display inherent phenotypic and epigenetic plasticity resulting in complex heterogenic diseases. In this review, we discuss the key principles of deregulation of epigenetic processes that shape this disease evolution. We consider measures to define and quantify clonal heterogeneity, combining information from recent studies assessing mutational, transcriptional and epigenetic landscapes at single cell resolution in myeloid neoplasms (MN). We highlight the importance of integrating epigenetic and genetic information to better understand inter‐ and intra‐patient heterogeneity and discuss how this understanding further informs evolution and progression trajectories and subsequent clinical response in MN. Under this topic, we also discuss efforts to identify mechanisms of resistance, by longitudinal analyses of patient samples. Finally, we highlight how we might target these aberrant epigenetic processes for better therapeutic outcomes and to potentially eradicate LSCs.

## Graphical abstract

In leukaemic stem cells (LSCs), aberrant epigenetic mechanisms converge and establish intratumoral heterogeneity, supporting leukaemic growth. These aberrant mechanisms lie directly downstream of epigenetic mutations or indirectly relate to metabolic and cell‐extrinsic signalling. Besides initiating and maintaining the LSC state, these aberrant mechanisms also provide potentially targetable vulnerabilities to help eradicate LSC and cure leukaemias.

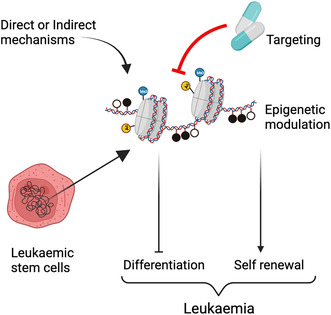

**Abbreviation**
2HG: 2‐hydroxyglutarate
AA: amino acid
ADC: antibody‐drug conjugates
AML: acute myeloid leukaemia
AMPK: AMP‐activated protein kinase
BCAT1: branched‐chain amino acid (BCAA) transaminase 1
BET: bromo‐ and extra‐terminal domain
BiTEs: bispecific antibodies
CH: clonal haemopoiesis
CML: chronic myeloid leukaemia
CUT&TAG: cleavage under targets and tagmentation
DNMT: DNA methyltransferase
DTA: DNMT3A, TET2 and ASXL1
FA: fatty acid
GMP: granulocyte monocyte progenitor
GRC: gene regulatory complex
HDACi: histone deacetylase inhibitor
HMA: hypomethylating agents
HSC: haematopoietic stem cell
IDH1/2: isocitrate dehydrogenase enzymes 1/2
LIC: leukaemia stem or initiating cell
LSC: leukaemic stem cell
MDS: myelodysplastic syndrome
MN: myeloid neoplasm
MPN: myeloproliferative disorder
PDX: patient derived xenotransplant
PRC1/2: polycomb repressive complexes 1 and 2
ROS: reactive oxygen species
SAM: S‐adenosyl‐methionine
SCID: severe combined immunodeficiency
TCA cycle: tricarboxylic acid cycle
VAF: variant allele frequency
α‐KG: alpha keto‐glutarate

## 1 Introduction

Leukaemia stem cells (LSC) are the critical unit of growth and selection within haematological malignancies and are responsible for the initiation and further evolution of myeloid neoplasms (MN). As such, they dictate the eventual phenotype of the MN and are responsible for its potential progression and relapse following therapy. This review will provide a broad overview of how epigenetic mutations and epigenetic processes aberrantly shape this evolution, both mechanistically and clinically, and how we might target these corrupted epigenetic processes for therapeutic gain, thus improving outcomes in MN.

## 2 The probabilistic continuum of LSCs in MN

**Fig. 1:**
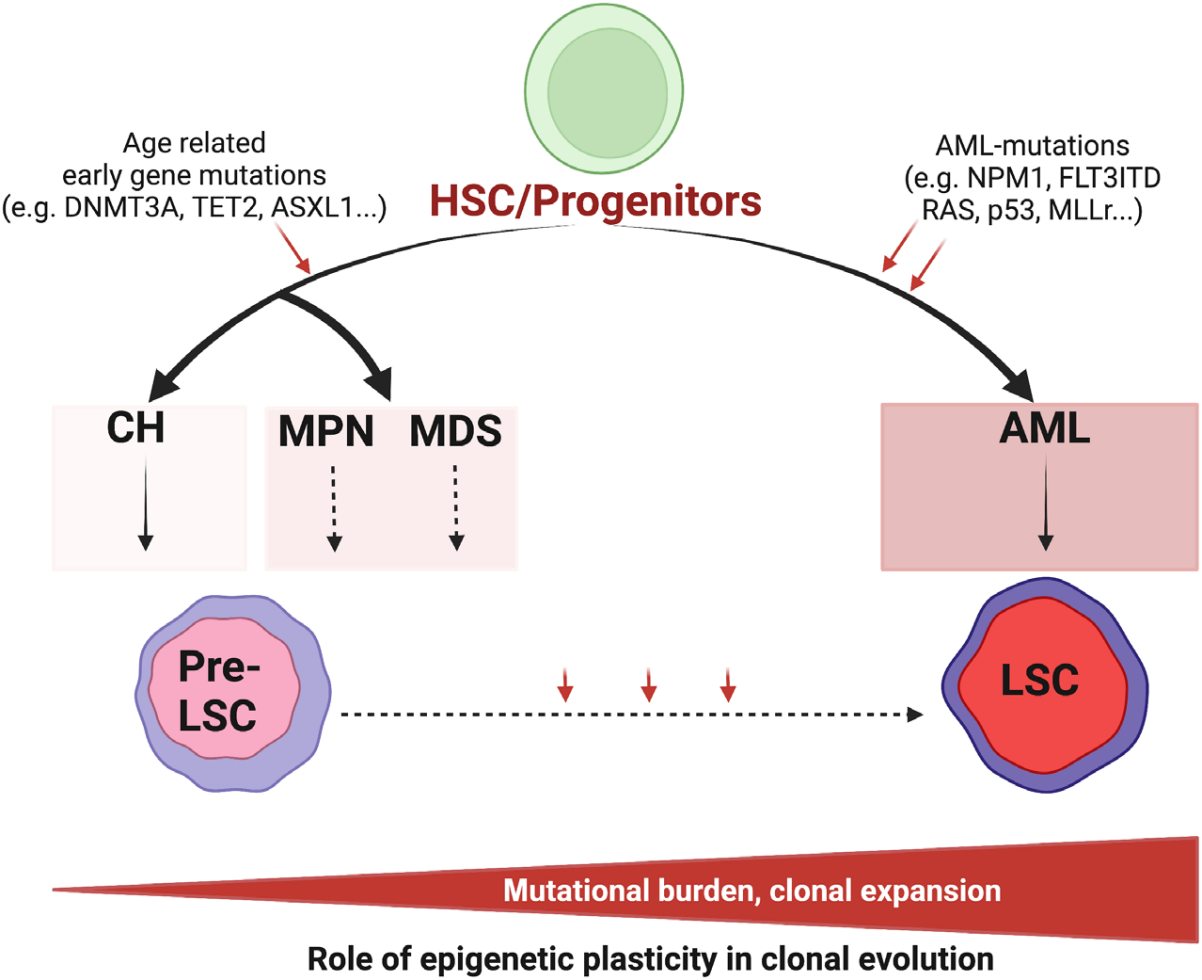
The continuum of Myeloid Neoplasia. This schematic highlights that LSC and pre‐LSC are generated from normal Haematopoietic Stem and Progenitor cells via the acquisition of mutations and likely other non‐genetic aberrations. The LSC may further evolve and expand along the continuum of Myeloid Neoplasms (MN) from clonal haematopoiesis (CH) through precursor Myeloproliferative (MPN) and Myelodysplastic (MDS) stages to AML, with this progression associated with an acquisition of mutations and epigenetic aberrations. We speculate that epigenetic plasticity facilitates this transition. Red arrows represent genetic or epigenetic hits acquired during disease evolution. Created with BioRender.com.

Myeloid neoplasms comprise a spectrum of disorders (Fig. 1). Their continuum of phenotypes range from generally chronic, indolent disorders, characterised by overproduction of mature myeloid cells, such as the myeloproliferative disorders (MPN) and chronic myeloid leukaemia (CML), through more aggressive forms with increasing bone marrow failure phenotypes and higher medullary blast counts, myelodysplastic syndromes (MDS), to overt acute myeloid leukaemia (AML), a highly aggressive, often fatal malignancy characterised by overproduction of primitive blast cells and severe bone marrow failure. These phenotypes are linked, in that the natural history of MPN, CML and MDS leads to transformation to AML in a proportion of cases and that they share a number of common mutations, particularly those altering epigenetic regulators such as the DNA methyltransferase 3A (*DNMT3A*), the DNA‐demethylase *TET2* and the BAP1‐interacting factor *ASXL1* [1]. Recently, massive parallel sequencing of normal individuals lacking a haematological diagnosis has added a further layer of complexity to this evolutionary model [2]. These studies have demonstrated that cancer‐defining mutations recurrent in MN, especially AML, such as *DNMT3A*, *TET2* and *ASXL1* (so‐called DTA mutations), are present in these otherwise normal individuals. This phenomenon has been termed Clonal Haemopoiesis (CH) and a plethora of studies have demonstrated a significantly increased relative risk of, but not inevitable, development of MN. This defines CH as a potentially pre‐malignant state and it is likely that the mutant Haematopoietic stem cell (HSC) acts as a potential “pre‐LSC”.

As progression is not inevitable, the trajectories for evolution of CH and MN are not deterministic, rather they are probabilistic for further evolution. However, the factors that facilitate further evolution are currently unknown and a critical research topic is to elucidate the nature of these factors, driven by the promise of earlier intervention and possibly the prevention of the development of MN or the progression to AML. Due to the central role of epigenetic mutations across the CH‐MN continuum and the demonstration of epigenetic alterations at multiple levels across this continuum, aberrant epigenetic processes are obviously key to these transitions, and these will be discussed further below.

## 3 LSC characteristics and their epigenetic determination

### 3.1 Defining the LSC: function over form

Leukaemia stem or initiating cells (LIC) can only be properly defined in functional assays; for human LSC, they are the cells capable of initiating a phenotypically identical disease to that within the patient in an immunocompromised mouse system. They were first described almost three decades ago in pioneering work by the Dick laboratory [3], where AML progenitors were shown to generate disease in SCID mice at low frequency, with enrichment of disease transfer demonstrated for transplantation of the primitive CD34^+^/CD38^−^ fraction. Since then, significant improvements in the degree of immunocompromise in models and the expression of human cytokines within them, aimed at overcoming a lack of cross‐reactivity between species, have greatly increased the efficiency of these patient‐derived xenotransplant (PDX) models and they are now routinely used for mechanistic and translational studies.

However, trying to define an LSC solely by its surface phenotype has proven elusive. Whilst the majority of LSC are enriched within the CD34^+^ compartment, up to ~ 25% of AML cases are CD34‐, and LSC activity can be demonstrated for these tumours. Additionally, in more immunocompromised strains, LSC activity can also be demonstrated in the CD34^+^/CD38^+^ progenitor compartment. Therefore, a plethora of further surface candidates have been studied, including (but not limited to); CD123, CD33, CLL1 (CD371), CD93, CD96, CD99, ILRAP‐1, TIM3, GPR56, CD47 and CD25, with each found to enrich for LSC activity [4, 5, 6]. Mechanistically, the aberrant regulation of these surface markers likely reflects the stalled differentiation trajectories seen in AML and/or the aberrant transcriptional/epigenetic landscapes present.

Expression and, equally, lack of expression of cell surface markers reflects, at least in part, the effect of gene regulation, a process critically controlled by epigenetic regulation. These cell surface markers have diverse cellular roles which co‐ordinate the external stimuli to the internal cellular environment. Inhibitory immune checkpoint receptors, such as TIM‐3 and PD‐L1, have been associated with poor survival and are implicated in hypomethylating agent resistance [7, 8, 9]. Furthermore, epigenetic gene silencing of activating immune receptor genes in AML has been observed; the expression of which can be shown to be rescued upon treatment with HDACi [10].

However, to summarise a vast number of studies, no single marker has been shown to be uniformly present on all LSC across patients, none are solely indicative of LSC activity and markers may vary with disease phase and even within patients. These findings emphasise inter‐ and intra‐patient heterogeneity (see below), but obviously also have therapeutic implications (also see below) in that no one marker provides a target present across all LSC. Moreover, although some of these markers are confined to cells lower in the haematopoietic hierarchy than normal HSCs, many are present at varying levels on HSC, thus providing a potentially narrower therapeutic index for their targeting.

### 3.2 Aberrant metabolism in LSCs

A number of recent studies have also identified alterations of metabolic processes between normal HSC and LSC. Although both HSC and LSC are associated with relatively lower levels of reactive oxygen species (ROS) than their progeny, LSC have been shown to be more dependent upon oxidative phosphorylation (OXPHOS) for their energy requirements than HSC, which preferentially use glycolysis [11]. These two processes also potentially utilise different fuel sources; glycolysis is dependent upon glucose metabolism; however, OXPHOS may also utilise amino acid (AA) and fatty acid (FA) metabolism. LSC have been shown to be dependent upon AA metabolism for their increased OXPHOS [12]. Correlative transcriptomic and quantitative proteomic analyses have also demonstrated an enrichment in LSCs for pro‐OXPHOS proteins such as CRIP2, CD26 and many fundamental members of the electron transport chain [13]. Additionally, the authors demonstrated key LSC metabolic characteristics visible across different AML genetic subtypes, indicative that therapies targeting these central pathways may be largely effective across multiple AML subtypes.

### 3.3 Aberrant metabolism and epigenetics in LSC

Altered LSC metabolism is intimately linked to aberrant chromatin state. DNA and chromatin modifiers utilise intermediate metabolites such as S‐Adenosyl‐Methionine (SAM) and Acetyl‐Co‐A as methyl and acetyl donors, respectively and metabolites such as alpha keto‐glutarate (αKG) and FAD as co‐factors in histone and DNA demethylation and NAD^+^ in histone deacetylation. Therefore, epigenetic states are extremely sensitive to fluctuations in these metabolite levels [14]. These processes are often altered by MN mutations, such as mutations in the cytosolic and mitochondrial isocitrate dehydrogenase enzymes, *IDH1* and *IDH2*, respectively. The *IDH1* and *IDH2* mutations generate enzymes with neomorphic functions that further catalyse the reduction of αKG to 2‐hydroxyglutarate (2HG). 2HG functions as a competitive inhibitor of αKG‐dependent dioxygenase enzymes, such as TET2, that demethylate histones and DNA, with these changes subsequently altering gene transcription. In addition, LSCs demonstrate an increased activity of the branched‐chain amino acid (BCAA) transaminase, BCAT1, another critical regulator of αKG levels within LSC. BCAT1 transfers alpha‐amino groups from BCAA to αKG to generate glutamate, with increased BCAT1 activity in LSC associated with αKG depletion, phenocopying *IDH1/2* and *TET2*‐mutant gene expression patterns and cellular phenotypes. Thus, aberrant metabolic states are inextricably linked to altered epigenetic states within LSC and may provide therapeutic vulnerabilities [15]. The AMP‐activated Protein Kinase (AMPK) signalling pathway further demonstrates the crosstalk between metabolism and epigenetics. This central metabolic regulator has been found to have both tumorigenic and tumour suppressive functions in a variety of cancers. Within multiple AML models, AMPK activation has been shown to protect LSCs from metabolic stress and maintain their leukaemogenic potential [16]. Furthermore, AMPK mediated modulation of acetyl‐coenzyme A (CoA) homeostasis and reduced acetylation affected BET protein recruitment, suggesting a direct influence of aberrant metabolism on epigenetic landscape [17]. Interestingly, AMPK has also been shown to alter epigenetic state via the phosphorylation of epigenetic modifiers (including DNMT1, EZH2 and TET2) [18, 19, 20]. The link between AMPK and direct modulation of epigenetic factors has not been demonstrated directly in LSCs, however future studies could establish whether high levels of AMPK in LSCs regulate metabolic stress as well as modulate the epigenome to maintain leukaemic potential.

### 3.4 Altered epigenetic and transcriptional states within LSCs

**Fig. 2:**
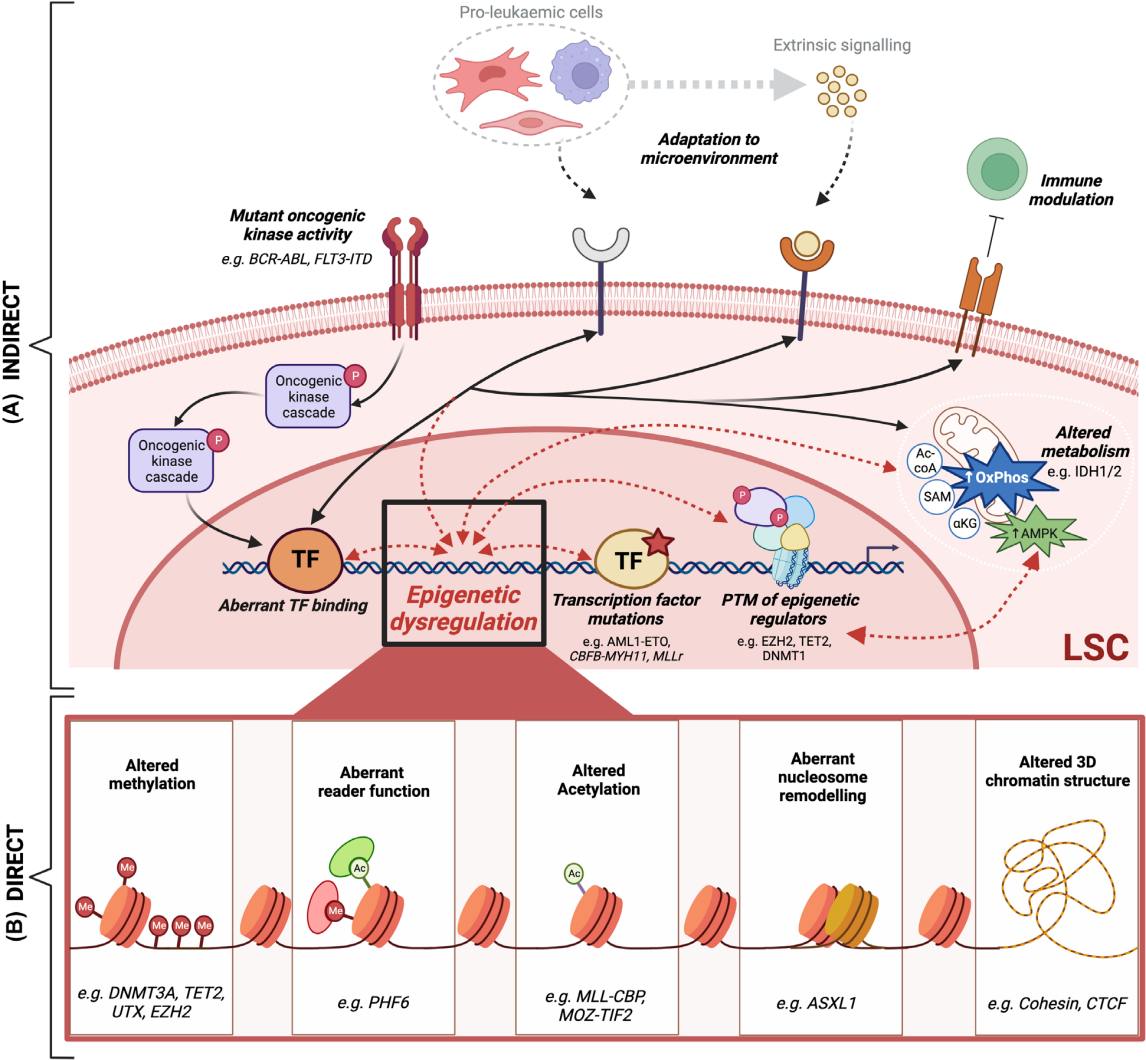
Alterations in Myeloid Neoplasia are centrally mediated through altered epigenetics. This schematic demonstrates cell extrinsic (microenvironmental and immunomodulatory) and intrinsic (oncogenic signalling, metabolism and TF/Epigenetic mutations) factors that combine to alter the epigenetic landscape and generate leukaemia gene expression programmes. Upper panel of the figure shows examples of non‐genetic stimuli (indirect) impacting on epigenetic dysregulation; lower panel shows epigenetic dysregulation mediated by mutations in epigenetic regulators (direct). Examples of epigenetic function are given in the lower part of the figure. Created with BioRender.com.

During haematopoiesis, multiple mature effector cells with highly disparate functions are generated from a single cell type, the HSC. This requires exquisite orchestration by gene regulatory complexes (GRC), with cell fate decisions led by lineage‐specific transcription factors. Recently, we have also shown the specific requirement for individual epigenetic regulators, as part of these GRC, in these individual decisions [21]. Together, the complexes co‐ordinately remodel the epigenetic landscape and mould cell‐type specific transcriptional programmes which give the mature cells their separate identities and phenotypes. It is hardly surprising therefore that these processes are hijacked during the stepwise evolution of malignancy. A proportion of these epigenetic regulators are directly mutated; the DNA demethylase *TET2* and the *IDH1/2* genes have already been mentioned. Others such as inactivating mutations of the gene encoding the *de novo* DNA methyltransferase *DNMT3A*, histone lysine 27 methyltransferase *EZH2*, that generates specific patterns of DNA or H3K27 hypomethylation, or truncating mutations of the *ASXL1* gene that lead to activation of the BAP1 deubiquitinase complex [22], generate transcriptional programmes that facilitate myeloid cell transformation. In addition, other chromatin factors are dysregulated at the level of transcription, and we also suspect that the same or other factors are the substrates for aberrant oncogenic signalling, a common feature of MN. The presence of distinct epigenetic landscape in LSCs from *MLL (KMT2A)‐*rearranged (*MLLr*) leukaemias, for example, aberrant H3K4me2/3 patterns and/or EP300 associated pervasive enhancer malfunction, separate LSC from non‐self‐renewing progeny of leukaemia cells. Of note the “LSC fraction” used in these studies were relatively broad, comprising the cKit^+^ compartment [23, 24]. With updated knowledge of LSC surface markers, future studies could further focus on the apex of the AML hierarchy to elucidate deeper mechanistic insights. Undoubtedly, these findings could be utilised to target regulatory enhancer axes, and/or key epigenetic enzymes such as DOT1L or LSD1 in LSC from *MLLr*‐or other leukaemia subtypes (discussed further in therapy section). Taken altogether, there is abundant evidence for aberrant epigenetic activity, via direct or indirect mechanisms, in LSCs (as illustrated in Fig. 2).

## 4 LSC heterogeneity and epigenetic plasticity: substrates for disease progression and drug resistance

Disruption in the homeostatic balance of epigenetic states due to genetic, metabolic or environmental stimuli may result in a more permissive/plastic chromatin state. We and others propose that this epigenetic plasticity will allow adaptive transcriptional changes that are propagated through mitosis resulting in the expansion of a new clone due to increased fitness [25]. Besides the aforementioned examples, that generate an epigenetically plastic state due to gene mutations or translocations involving chromatin modulators, epigenetic plasticity could also result from non‐genetic causes. For instance, we have shown that loss of *Ezh2* (non‐mutated) contributes towards leukaemia initiation in combination with *AML1/ETO*, where H3K27me3 deposition is altered, providing a plastic and permissive epigenetic state for oncogenes to further fuel malignant progression [26]. Moreover, loss of EZH2‐ or DNMT3A‐function is shown to induce chemoresistance in AML [27, 28]. Furthermore, aberrant DNA methylation signatures have been used to define AML‐subtypes and in the majority of cases these were independent of underlying genetic event [29]. Alternatively, aberrant DNA methylation‐induced alterations in 3D genome topology, due, at least in part, to defective binding of CTCF, have also been described in AML (illustrated in Fig. 2) and discussed later in this review.

**Fig. 3:**
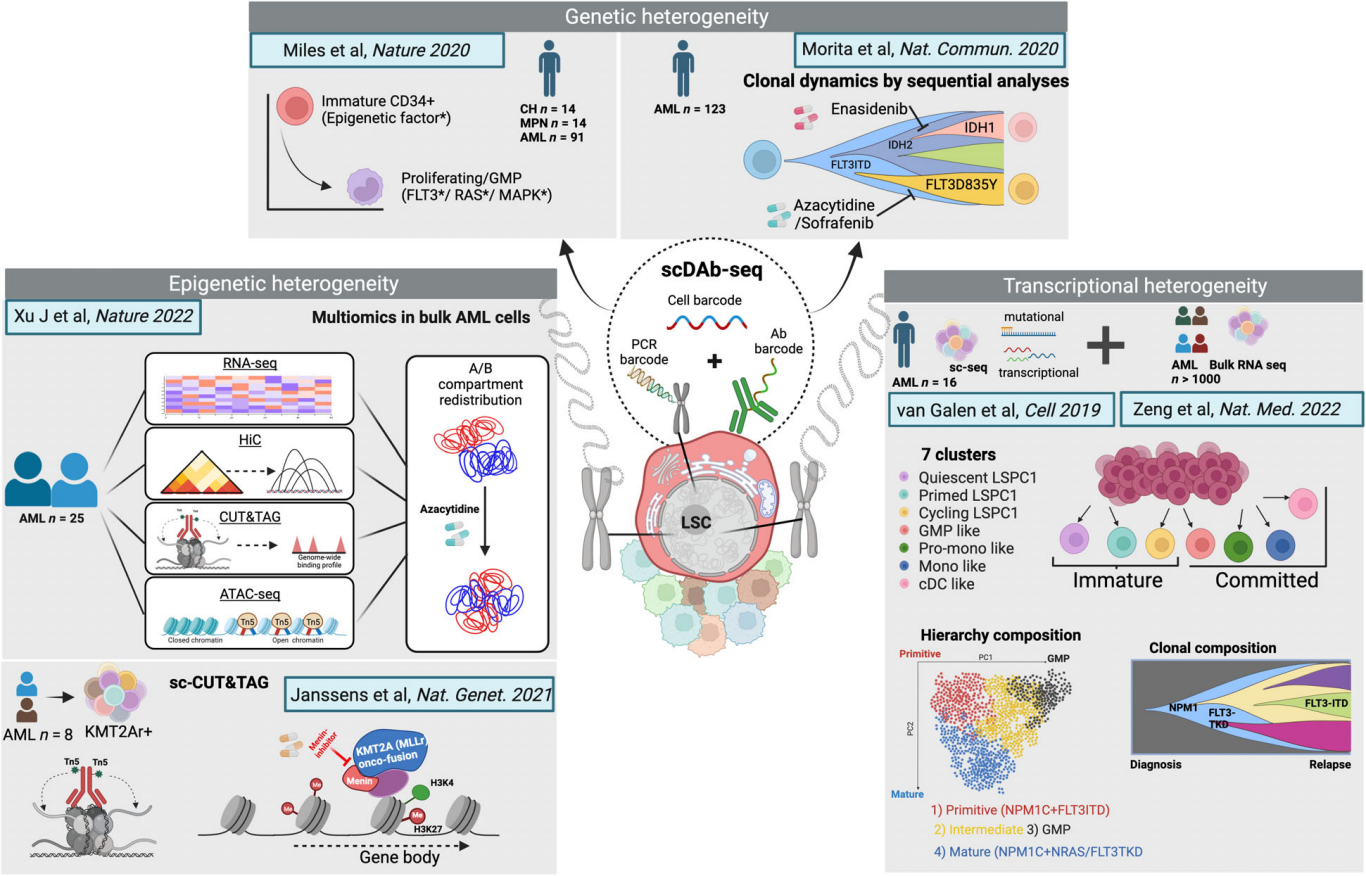
Recent studies highlight LSC heterogeneity at single cell resolution. Illustrative studies, discussed further in the text, have demonstrated the degree of heterogeneity that exists within the LSC compartment, providing insights into the role of LSC heterogeneity in disease evolution, prognostication and the development of resistance and relapse. Created with BioRender.com.

Early experiments assessing bulk genetic and genomic characteristics, as well as immunophenotyping experiments at single cell resolution had demonstrated heterogeneity between and within AML patients. In addition, a small number of longitudinal studies, serially examining the same characteristic iteratively within individual patients have added a temporal dimension to this heterogeneity and have also highlighted the selective pressures of therapies in shaping the evolution of disease [22]. However, recent studies conducted predominantly at single cell resolution and across epigenetic and/or transcriptional readouts have added significant granularity to the degree of heterogeneity. We will highlight some of these below (illustrated in Fig. 3) and summarise their implications for the evolution and progression of MN, as well as the challenges that this heterogeneity presents for the therapeutic eradication of LSC.

### 4.1 Genetic heterogeneity

Massive parallel sequencing of bulk tumours can quantitatively infer the frequency with which a gene is mutated within the population of cells. This variant allele frequency (VAF) can therefore be calculated for the multiple mutations required to drive individual aggressive tumours such as AML and can dynamically quantitate the size of the premalignant or malignant clone in CH and MPN and MDS. The mutational burden is usually less in CH, MPN and MDS, with mutant clones often bearing just a single mutation and existing in a mosaic pattern alongside “normal” HSC that lack mutations. These “snap‐shot” bulk studies, usually taken at diagnosis, along with longitudinal studies from relapse or transformed AML, have demonstrated that multiple clones exist within MN and that these can evolve over time and particularly under the selective pressures of chemotherapy. More recently, single cell DNA‐sequencing studies of specific mutations combined with surface immunophenotyping (scDNA‐Ab‐sequencing or scDAB‐Seq) have resolved this evolution at the single cell scale. In a seminal study, Miles et al resolved the clonal architecture of 119 patients with CH, MPN and AML [30]. They found that scDAB‐Seq accorded with bulk VAF, but was more sensitive, identifying rare mutations not picked up by bulk sequencing. Assessing clonal architecture, they could infer that AML was usually initiated by a mutation in an epigenetic regulator (such as *IDH1/2, TET2* or *DNMT3A*) or transcription factor. They suggested these mutations to induce epigenetic remodelling and alter differentiation, generating a pool of proliferating stem‐like cells that favoured the acquisition of mutations affecting genes encoding for signalling molecules, such as *FLT3‐(ITD)* or *RAS*. This was further supported by the observation that mutations in epigenetic or transcription factors were confined to the immature CD34^+^ cells, whereas mutations in MAPK pathways signalling molecules were more abundant in the mature CD11b^+^ compartment. Moreover, the consequential effect of treatment on clonal heterogeneity could also be identified by scDAb‐seq [31]. Specifically, sequential analyses of individual patients revealed the emergence of resistant *FLT3‐D835Y* (FLT3 Tyrosine kinase domain – TKD) clones after Azacytidine/Sorafenib treatment and loss of sensitive *FLT3‐ITD* and *FLT3‐D835E* clones. Other examples demonstrated the emergence of an IDH1‐mutant clone upon combination treatment with the IDH2 inhibitor Enasidenib and Azacytidine, or the replacement of a (sensitive) *FLT3*‐mutant clone by a (resistant) RAS mutant clone after FLT3‐inihibitor treatment. Therefore, the effects of therapy on clonal composition can be very sensitively followed genetically at single cell resolution and suggest obvious patterns of genetic resistance.

### 4.2 Transcriptional heterogeneity and LSC signatures

More recently, a number of studies have refined and expanded our understanding of LSCs and intra‐patient heterogeneity in AML, using single cell RNA‐sequencing combined with mutational analyses to link genotype with transcriptional phenotype. A longitudinal study from van Galen and Bernstein who sampled 16 AML patients at diagnosis and during treatment and used a machine learning classifier, revealed the existence of six malignant clusters along the HSC to myeloid differentiation axis, with the abundance of each cluster varying widely between AML cases with different genotypes [32]. Several important observations were made, including the functional association of *FLT3‐ITD* positive clones with a primitive cell predominance; suggesting that *FLT3‐ITD* blocks differentiation or transforms a more primitive progenitor, whereas *FLT3‐TKD* subclones in the same tumour primarily contained more differentiated cells.

Revisiting this scRNA seq data and the cell state/differentiation spectrum framework, Zeng and Dick have proposed a methodology for understanding AML LSC heterogeneity and the use of the cellular hierarchy to predict drug response in individual AML patients [33]. In a two‐step process, first reanalysing scRNA seq data from 12 AML patients, they defined 7‐AML subpopulations ranging from immature to more differentiated cell states. In the second step, the abundance of each of these cell types was estimated in more than 1000 AML patient bulk RNA seq datasets, using an R‐based algorithm CIBERSORTx. This approach deconvoluted bulk gene expression data using the single‐cell framework to generate a characteristic “hierarchy composition” and assign each sample to one of four groups—primitive stem‐like leukaemia cells (Primitive), granulocyte monocyte progenitor‐like leukaemia cells (GMP), leukaemia cells of mature phenotype (Mature) or intermediate composition (Intermediate), using two principle components; (a) a continuum of primitive‐to‐GMP‐enriched and (b), a continuum of primitive‐to‐mature‐enriched leukaemic cellular hierarchies. Of note, the primitive‐GMP axis appears to reflect cytogenetic abnormalities, whereas the primitive‐mature axis was more associated with genetic driver mutations and their combinations.

Interestingly, different driver mutations in the same gene could have different consequence on cellular hierarchies. For example, *DNMT3A R882* mutations may be more permissive of AML differentiation than other DNMT3A mutations. Additionally, *RAS* mutations favoured differentiation, whereas *IDH* mutations were associated with an early differentiation block. This framework could also capture clonal evolution, where changes in the hierarchical assignment often changed upon relapse, often with primitive expansion at relapse. In assessing mutational synergies, in the case of the *NPM1C* mutation, the co‐occurrence of *FLT3‐ITD* with *NPM1C* usually generated Primitive hierarchies, whereas mutant *NRAS* or *FLT3‐TKD* with *NPM1C* generated Mature hierarchies. In addition, *FLT3‐ITD* alterations were recurrently acquired at relapse, whereas *NRAS* and *FLT3‐TKD* alterations were recurrently lost at relapse in the context of NPM1‐mutant AML. However, most importantly, the shifts in hierarchy composition occurred in the absence of clear genetic changes, as has been seen with bulk studies [34], once again pointing to the importance of other non‐genetic factors, such as epigenetic remodelling, in driving disease progression.

Further reflecting the prognostic/predictive nature of the hierarchy within AML, various transcriptional scores have been devised to aid clinical decision‐making. The LSC17 score, devised from those genes whose expression is enriched in LSC‐enriched fractions in comparison with the fraction lacking LSC activity, is currently a certified clinical assay. This score was further improved upon in the Zeng study, to generate the LinClass‐7 score which could more accurately classify prognostic outcome in large AML studies [33].

### 4.3 Epigenetic heterogeneity

Epigenetic heterogeneity within the pre‐LSC and LSC has also been studied at the single cell level. A study from the Landau group, utilising single cell multiomics (capturing genotype, transcriptomics and methylome), analysed samples from individuals with *DNMT3A R882*‐mutated CH [35]. This work showed a myeloid‐lymphoid bias for the *DNMT3A R882*‐mutated cells, demonstrating the requirement for precise methylation patterns in determining differentiation. Moreover, these studies outlined an increased pool of immature myeloid progenitors with mega‐erythroid priming that expressed dysregulated lineage markers and increased LSC markers, that may acquire further lesions and fuel pre‐LSC evolution and disease progression.

In AML, a recent study from Xu and colleagues performed multiomics [HiC, RNAseq, ATACseq, Cleavage Under Targets and Tagmentation (CUT&TAG) for CTCF, H3K27ac, H3K27me3] from the same AML samples in 25 AML samples and 7 normal donors, combined with whole genome sequencing to analyse copy number‐ and structural variations that could further impact the epigenome [36]. This study provides an extremely rich dataset that could be used further to establish a framework or reference map of AML epigenetic landscape, ultimately revealing AML subtype‐specific alterations in the 3D genome. These alterations highlighted the association between alterations in 3D genome topology and aberrant DNA methylation, related to the binding of the major enhancer‐promoter loop anchor protein CTCF. The therapeutic implications of this understanding is further discussed in the therapy section in this review. Chromatin factor binding and histone modification profiling have also recently been profiled in AML at both low‐input, bulk and single cell resolution. In a set of eight primary AML samples and some exemplar AML cell lines with rearrangements of the *KMT2A* (*MLL1*) locus (*MLLr*), Janssens, Henikoff and colleagues, successfully applied an sc‐CUT&TAG approach to map differential genomic binding of KMT2A (wt or mutated) together with profiles for the H3K4me3 and H3K27me3 histone modifications [37]. Importantly, a subset of KMT2A oncofusion‐binding sites were demonstrated to be marked by bivalent (H3K4me3 and H3K27me3) chromatin signatures and were associated with cell‐to‐cell heterogeneity, suggestive of increased lineage plasticity. Additionally, therapeutic vulnerabilities to novel targeted therapies were identified; aberrant enrichment of H3K4me3 in gene bodies, that has been associated with transcriptional upregulation of these genes bound by KMT2A oncofusions, was shown to be targeting by Menin inhibitors. Taken together these studies highlight mechanisms of epigenetic dysfunction and link both standard‐of‐care and novel therapeutics with reversion of aberrant epigenetic patterns.

## 5 Potential therapeutic strategies to target LSC

**Fig. 4:**
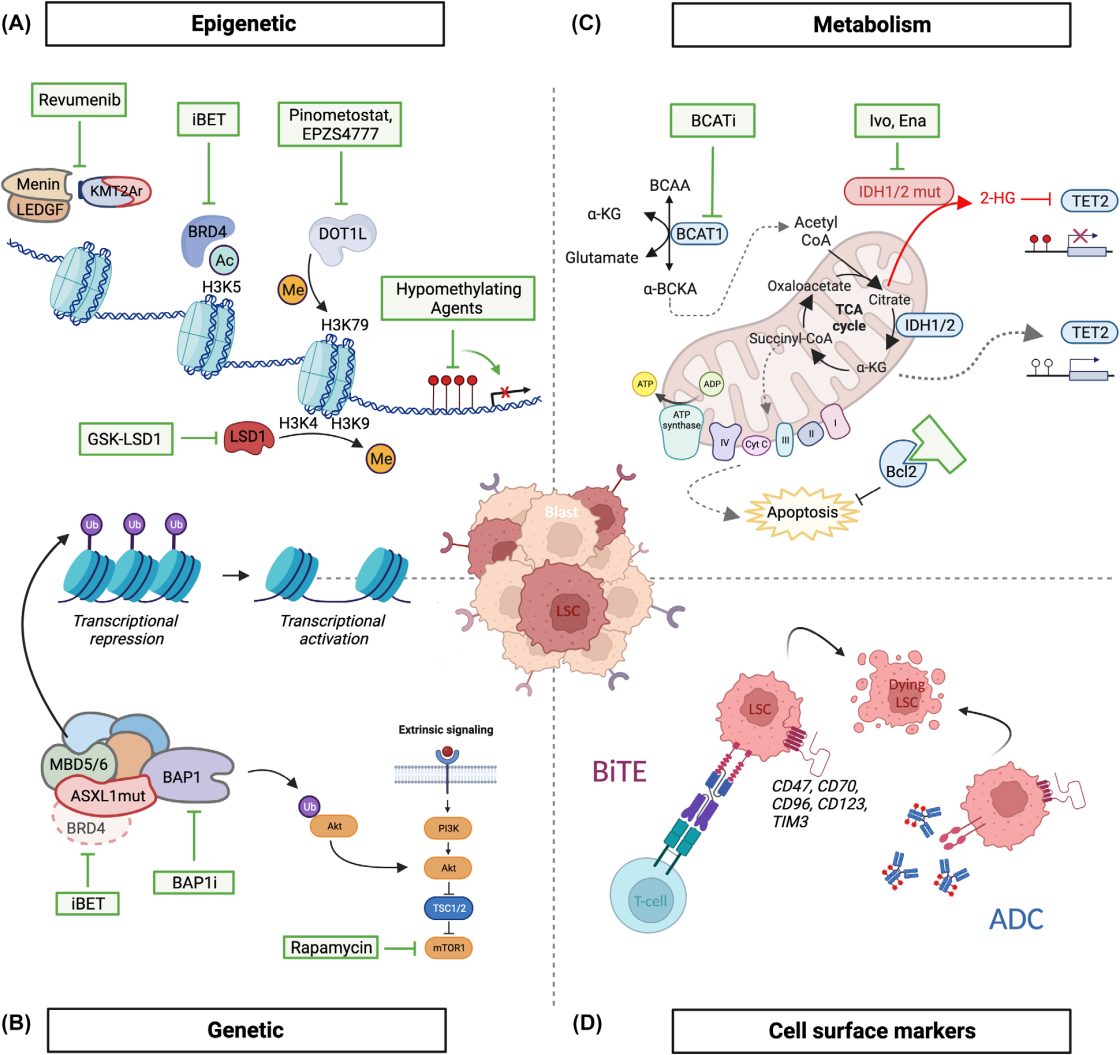
Targeting LSC. This schematic shows four promising areas for targeting LSC that are discussed further in the text: Epigenetic (A), Genetic (B), Metabolism (C) and via Cell surface markers (D). Created with BioRender.com.

As pre‐LSC and LSC are the drivers of disease initiation and evolution and provide the reservoir for relapse and the development of resistance within MN, they are the critical cellular targets within these diseases. Although targeting pre‐LSC to either delay or actually prevent the development of overt MN remains an ambition and an area of intense interest and study, we currently lack the knowledge of mechanisms of CH evolution and pre‐LSC biology. We will therefore focus on potential means to target LSC in AML in this section (see also Fig. 4).

### 5.1 Direct targeting of epigenetic regulators

As detailed in previous sections of this review, our understanding of aberrant epigenetic processes mediated by, or downstream of mutations in AML has significantly improved over recent years. This has allowed the development of therapeutic strategies designed to correct epigenetic aberrations by specifically targeting enzymes and proteins aberrantly involved in these processes.

#### 5.1.1 Hypomethylating agents (HMA)

In this regard hypomethylating agents (HMA), such as 5‐azacitidine (5‐aza) and decitabine, have been widely used in the clinic over the past 20 years, notably in the treatment of MDS and AML, although it is likely that these agents have additional mechanisms of action outside of their role in methylation (Fig. 4A). A study by Xu and colleagues, demonstrated that treatment of AML with HMA partially restored the chromatin structure towards the normal configuration, including reverting/switching genome compartment from B to A and dissociating AML‐specific chromatin loops. These important observations suggest that treatment with an HMA may achieve therapeutic efficacy, at least in part, through the restoration of normal chromatin architecture and epigenetic patterning [36]. Use of these agents aims to reactivate critical genes silenced by methylation and thus to revert cell proliferation and trigger differentiation. Cancer is generally characterised by global hypomethylation, with local hypermethylation at promoters and other regulatory elements which combine to alter gene expression. By covalently binding the maintenance DNA methyltransferase, DNMT1, leading to its loss of function and degradation, low concentrations of HMA prevent the deposition of methyl group on newly synthesised DNA strands during replication. This allows for a passive loss of DNA methylation after each round of cell division, ultimately leading to the reactivation of cell cycle regulators and tumour suppressor genes, such as p14 and p15, which have been previously described in this context [38]. Used as single agents, HMA have demonstrated relatively modest effects. However, more promising effects have been achieved with the use of either of these drugs in combination with Venetoclax, a highly specific BCL2 inhibitor (see below), although the nature of this synergism is not known.

#### 5.1.2 Inhibitors of chromatin modifiers

As mentioned earlier in this review, epigenetic alterations are a major contributor to malignant transformation. They have therefore attracted lot of attention, leading to the development of specific “epigenetic therapies” (Fig. 4A). Numerous therapeutic agents have been developed against a plethora of epigenetic regulators. Amongst them, Pinometostat, a first‐in‐class small‐molecule inhibitor targeting the H3K79 methyltransferase DOT1L has been used in a phase 1 clinical trial in MLL leukaemias [39]. Although reduction of H3K79 methylation was observed in all patients, the degree varied across patients and did not correlate with Pinometostat plasma concentration. These results may, in part, be explained by the poor pharmacokinetics of Pinometostat and/or the interdependency between DOT1L and BRD4 in this type of leukaemia. In this regard, treatments combining Pinometostat and BET inhibitors may hold stronger therapeutic benefit [40]. In the context of leukaemia, H3K79me2 is known to maintain an open chromatin configuration to allow recruitment of transcriptional activators which subsequently induce leukaemic programmes. Consistent with this observation, MLL‐AF9 cells showed reduced chromatin accessibility upon DOT1L inhibitor (EPZS4777) treatment. Strikingly, inhibition of the lysine demethylase LSD1 using GSK‐LSD1 resulted in the opposite effect with a gain of chromatin accessibility [41]. However, dynamic loci shared very little overlap between treatments, suggesting multiple paths can abrogate self‐renewal and trigger myeloid differentiation to overcome the differentiation block uniformly seen in AML [41]. These results reinforce the idea that multi‐agent therapies are key to achieve sustained remission in patients. Many reversible and irreversible LSD1 inhibitors have been developed and are currently being evaluated in different settings in trials in AML and other malignancies [42, 43].

#### 5.1.3 Menin inhibitors

Menin, along with the LEDGF protein, is required for the recruitment of wild‐type KMT2A (MLL1), as well as KMT2A‐oncofusions, to regulate critical target genes. Small molecule inhibitors of the Menin‐KMT2A interaction (i.e. Revumenib) (Fig. 4A) have recently been developed that have shown promise in preclinical studies of AML driven by both KMT2A‐oncofusions and *NPM1C* mutations. Recent clinical studies with Revumenib have upheld this promise [44], demonstrating an overall response rate of 53%. As follow up in this study is limited, the duration of response cannot be estimated, but as with all previous single agent studies of epigenetic therapies in AML, it is unlikely to be durable. However, plans are already underway to combine Menin inhibitors with other chemotherapeutic agents to consolidate effects. Of note, for both Menin and IDH inhibitors (see below), the induction of differentiation and a so‐called “differentiation syndrome” can be seen, further implicating aberrant epigenetic regulation in LSC as driving the differentiation block apparent in AML.

#### 5.1.4 Inhibition of the BAP1/ASXL1 axis

ASXL1 plays a critical role in epigenetic remodelling by acting as a scaffold in chromatin complexes, such as the Polycomb Repressive Complexes 1 and 2 (PRC1/2). It also binds to BAP1, a component of the deubiquitinase (DUB) complex. BAP1 antagonises PRC1 repression by removing mono‐ubiquitin residue on H2AK119 (H2AK119Ub1), resulting in transcriptional activation [22]. *ASXL1* is frequently mutated in cancer and haematological malignancies and correlates with poor prognosis. The mutations appear to generate gain‐of‐function mutants that stabilise BAP1, increase its recruitment to chromatin and facilitate the removal of H2AK119Ub1, loss of H3K27me3 and the expression of pro‐leukaemic genes. Recently, BAP1 catalytic inhibitors have been developed that inhibit ASXL1‐driven leukaemic gene signatures and impaired tumour progression in an *in vivo* model of *ASXL1* AML [22]. Other studies have linked *ASXL1*‐mutated transformation to BET proteins [45] and to mTOR signalling [46], particularly at early stages of transformation and therapeutic targeting of these avenues may have efficacy in arresting early clonal expansions associated with *ASXL1* mutations (Fig. 4B).

### 5.2 Indirect epigenetic targeting via altering aberrant metabolism

Most therapies against aberrant metabolism in AML LSC have been directed against IDH1/2 and BCL2; however, a growing number are also targeting other processes such as OXPHOS, Ferroptosis, etc. We will limit our discussion to BCL2 and IDH1/2 inhibitors as the gathered evidence is strongest here (Fig. 4C).

#### 5.2.1 BCL2 inhibitors

BCL2 is the prototypic anti‐apoptotic member of the BCL‐2 protein family, which regulates apoptosis. Because it is aberrantly overexpressed in LSCs and contributes to maintain LSCs and myeloblast survival, BCL2 is an attractive therapeutic candidate [11]. Conceptually this novel approach relies on small molecule inhibitors that are structurally related to the BH3 domain, and competitively act in a dominant negative manner to trigger apoptosis through the release of pro‐apoptotic proteins (BAX and BAK) from their physical interactions with anti‐apoptotic partners. As such, Venetoclax is a highly specific BCL2 inhibitor, with no affinity for the other anti‐apoptotic proteins BCL‐XL and MCL1, making it more suitable for clinical use. Venetoclax has shown modest effects as a monotherapy [47], however, a phase 3 clinical trial on 431 participants with naïve AML assessing its efficacy in combination with 5‐aza demonstrated encouraging results. The overall survival was increased by 5.1 months and the rate of complete remission was higher (36.7% vs. 17.9%; *P* < 0.001) in the group receiving the dual treatment [48] compared to those receiving 5‐aza alone. Cumulative evidence has established that Venetoclax effects on LCSs are mediated through the inhibition of amino acid uptake, that subsequently reduces cellular metabolism via the TCA cycle and OXPHOS pathway, upon which LSC depend [12].

#### 5.2.2 IDH1/2 inhibitors

Mutations in the *IDH1* and *IDH2* genes occur recurrently in MN and are common in AML with a combined frequency of ~ 20% of all patients. Targeting their neomorphic enzymatic function, mutant IDH inhibitors have been designed, with Ivosidenib (IDH1) and Enasidenib (IDH2) recently approved by the Food and Drug Administration for the treatment of refractory or relapsed (R/R) AML patients with the relevant *IHD* mutations [49, 50]. Both molecules have shown promising, although not durable, efficacy as monotherapies with overall response rates of ~ 40% and are currently being evaluated with HMA and standard chemotherapy to improve their level of response and durability.

### 5.3 Novel therapeutic avenues exploiting aberrant cell surface markers

With the identification of cell surface markers that enrich for LSCs, antibody‐based therapies have been explored in AML treatment. These approaches primarily use mono‐clonal antibodies (mAb) that recognise the related antigens at the surface of the leukaemic cells to promote the immune response. These strategies have been further refined with the conjugation of cytotoxic molecules to mAb (Antibody‐drug conjugates, ADC) and bispecific antibodies (BiTEs), where one fragment recognises leukaemic antigens and the other one binds to and recruits the CD3 receptor of effector T‐cells (Fig. 4D). The potential therapeutic targeting of several markers are currently under clinical evaluation, although the lack of a single marker that is uniform in AML LSC and absent in normal HSC has hindered this field, as it has the development of CAR‐T cell therapy for AML. A number of potential antigens currently being clinically targeted include CD70 (targeted with the anti‐CD70 monoclonal Ab Cusatuzumab) [51] and CD123 (being targeted with the antibody‐drug conjugate, ADC, Tagraxofusp, and via the anti‐CD123 mAb Talacotuzumab). T cell immunoglobulin and mucin‐domain containing‐3 (TIM3) is another attractive candidate considering its high expression in AML LSC but not in normal HSC [52]. This marker selectively enriched for LSC in human AML samples, which then successfully reconstituted disease in recipient mice after transplant. This study provided evidence for targeting TIM3 in AML LSC by monoclonal antibody treatment [52]. Two clinical trials are currently investigating the efficacy of a high‐affinity humanised anti‐TIM3 monoclonal antibody (MBG453) in AML treatment (NCT03066648, NCT03940352). However, perhaps the most exciting studies are targeting CD47, a “don't eat me” signal that prevents phagocyte‐mediated consumption and is overexpressed in malignant cells and LSCs in particular. Based on promising preclinical studies, the humanised anti‐CD47 antibody (Magrolimab) has now entered clinical trials in various settings, in dual combination with 5‐azacitidine (NCT03248479), and in triple combination with 5‐azacitidine and Venetoclax (NCT04435691) [53].

In future, therapies might evolve (a) using multi‐specific antibodies or CAR‐T cells targeting two surface receptors on LSCs to increase specificity or (b) develop an ADC‐conjugate where epigenetic drugs are conjugated to antibodies that are specific to LSC‐surface markers.

## 6 Epigenetic plasticity and its role in the development of resistance

The major concern in MN is the continued progression of the diseases to a more advanced stage. Currently, there are a few, if any, therapies that alter the natural history of MPN and MDS to transform to AML and the vast majority of patients with AML will relapse with resistant disease, an often fatal scenario. As we have highlighted, epigenetic dysfunction and plasticity likely facilitate this trajectory. For AML, the major challenge in patient management is refractory or relapse disease. Relapse can clearly be attributed to LSCs, usually a clone that was present at the time of diagnosis. Paired diagnostic/relapse sample studies have been immensely helpful to unravel the biology of relapse. Tracking back mutations present at the time of relapse has provided direct evidence of a shared mutational profile between diagnostic and relapse samples, however as we have outlined above there is also often evidence of epigenetic evolution within the relapsed clone [34] and it is likely that the nature of resistance is mediated by both genetic and non‐genetic causes. These suggest a common cell of origin, supporting the idea that relapse arises from a resistant clone present prior to treatment, selected for by chemotherapy and that may or may not subsequently adapt again under the selective pressure of therapy. In addition, these studies also suggest that the selective pressure further favours selection of advantageous epigenetic features within this clone. A recent study has further extended these results, identifying two major cellular trajectories leading to relapse in AML. Relapse could either originate from a rare cell population featuring an HSPC‐like phenotype (relapse origin‐primitive ROp) or from a more committed population expressing a stem cell‐like transcriptional program (relapse origin‐committed, ROc) [54]. Regardless of the pattern involved in the process of relapse, these studies highlight the relevance of understanding the genetic and epigenetic bases of these trajectories, with this understanding to be utilised to optimise primary therapy and to prevent, or at least decrease the frequency of relapse and improve overall patient outcomes.

## 7 Conclusions

Exquisite regulation of transcriptional programmes is required to balance the dynamic requirements for a multitude of mature blood effector cells across the lifetime of an individual. Critical for this highly ordered regulation are epigenetic factors. Therefore, it is hardly surprising that the epigenetic process is subverted and corrupted along the long and probabilistic continuum of myeloid neoplasm formation. Mutational barcodes have given us further evidence of this inextricable link and enabling single cell studies performed longitudinally are elucidating the mechanisms of epigenetic dysfunction. Central to this subversion is the conversion of a normal HSC to a LSC and the further evolution of this LSC beyond AML diagnosis under the selective pressure of therapy. We need to further add to this knowledge base, characterise better the intermediate pre‐LSC and determine how therapy selects for resistant LSC. Our ultimate aim will be to utilise these data to halt evolutionary trajectories, preventing or slowing the progression of CH and MPN/MDS and decreasing the incidence of relapse and improving survival in those who do develop AML.

## Conflict of interest

The authors declare no conflict of interest.

## Author contributions

SA‐S, JB, NS and BJPH wrote the manuscript.
